# Weak Interactions in Dimethyl Sulfoxide (DMSO)–Tertiary Amide Solutions: The Versatility of DMSO as a Solvent

**DOI:** 10.1021/acs.jpcb.2c07155

**Published:** 2023-02-08

**Authors:** Camilla Di Mino, Adam J. Clancy, Andrea Sella, Christopher A. Howard, Thomas F. Headen, Andrew G. Seel, Neal T. Skipper

**Affiliations:** ‡ Department of Physics and Astronomy, University College London, Gower Street, LondonWC1E 6BT, U.K.; ¶ Department of Chemistry, University College London, 20 Gordon Street, LondonWC1H 0AJ, U.K.; § ISIS Neutron and Muon Source, Science and Technology Facilities Council, Rutherford Appleton Laboratory, Harwell Campus, DidcotOX11 0QX, U.K.

## Abstract

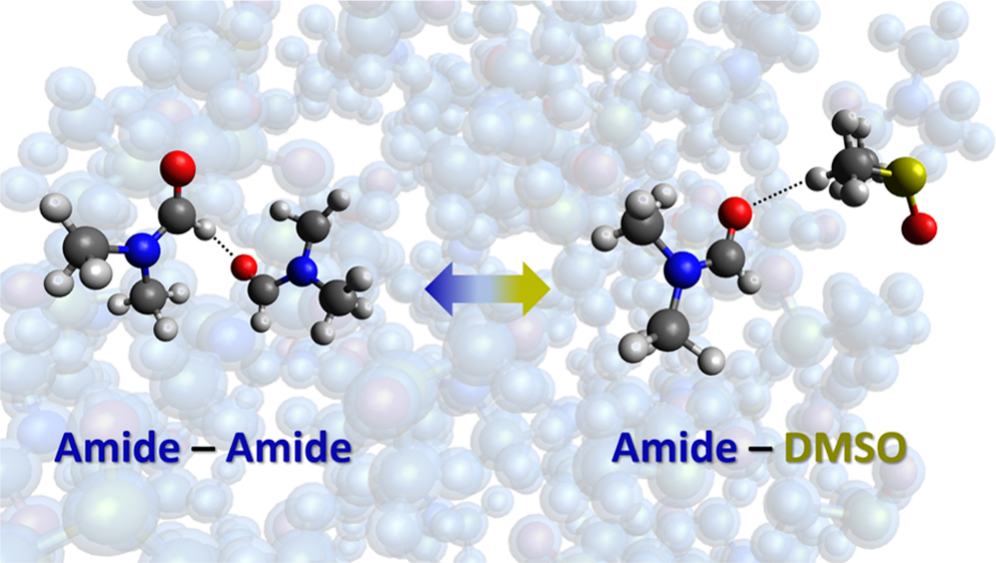

The structures of equimolar mixtures of the commonly used polar aprotic solvents dimethylformamide (DMF) and dimethylacetamide (DMAc) in dimethyl sulfoxide (DMSO) have been investigated via neutron diffraction augmented by extensive hydrogen/deuterium isotopic substitution. Detailed 3-dimensional structural models of these solutions have been derived from the neutron data via Empirical Potential Structure Refinement (EPSR). The intermolecular center-of-mass (CoM) distributions show that the first coordination shell of the amides comprises ∼13–14 neighbors, of which approximately half are DMSO. In spite of this near ideal coordination shell mixing, the changes to the amide–amide structure are found to be relatively subtle when compared to the pure liquids. Analysis of specific intermolecular atom–atom correlations allows quantitative interpretation of the competition between weak interactions in the solution. We find a hierarchy of formic and methyl C–H···O hydrogen bonds forms the dominant local motifs, with peak positions in the range of 2.5–3.0 Å. We also observe a rich variety of steric and dispersion interactions, including those involving the O=C–N amide π-backbones. This detailed insight into the structural landscape of these important liquids demonstrates the versatility of DMSO as a solvent and the remarkable sensitivity of neutron diffraction, which is critical for understanding weak intermolecular interactions at the nanoscale and thereby tailoring solvent properties to specific applications.

## Introduction

Polar aprotic liquids are widely used as solvents on both a laboratory and industrial scale, with important applications across a wide range of chemistry, biochemistry, and nanoscience.^1−3^ In this context, their relevant physicochemical properties include high dipole moments, high relative permittivities, and high boiling points, along with broad electrochemical stability windows when compared to their protic analogues.^4^ This combination of attributes makes these liquids highly effective for solvation of a wide spectrum of ions, small molecules, polymers, and nanostructures.^1,5−9^ For example, in electrochemistry, their inertness and ability to solvate both metal ions and polymeric coelectrolytes under highly reducing conditions are critical for battery function and stability.^2,3,10^ In addition, polar aprotic liquids provide a unique arena in which to study and tune the fundamental nature of weak intermolecular interactions, including C–H···O and C–H···π hydrogen bonds and both cyclic and acyclic π–π effects.

**Figure 1:**
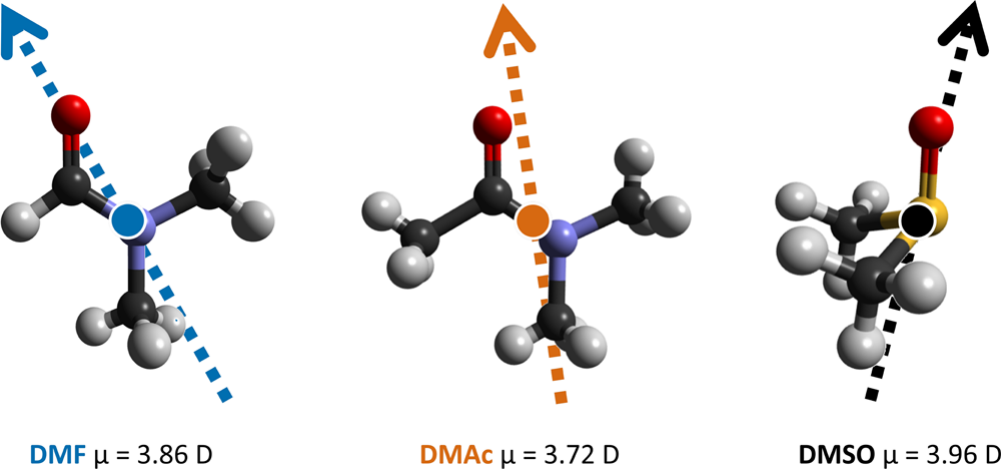
DMF, DMAc, and DMSO molecular models. The arrow indicates the direction of the molecular dipole, while the circle through which it passes highlights the center-of-mass (CoM) for each species.

Dimethylformamide (DMF, Me_2_NC(=O)H) and dimethylacetamide (DMAc, Me_2_NC(=O)Me) are the simplest aprotic amides, in which the proton donor (protic) N–H groups present in formamide (FA, H_2_NC(=O)H), *N*-methylformamide (NMF, MeHNC(=O)H), and *N*-methylacetamide (NMAc, MeHNC(=O)Me) are replaced by N–Me (Figure 1). The aprotic nature and the high dipolar character of these amides make them the ideal candidate for studying weak competitive interactions in the liquid state. Both DMF and DMAc are planar acyclic amides, where partial double bond character in the N–C=O framework arises from π electron delocalization that enforces the planarity of the molecule.^11^ DMF and DMAc have similar dipole moments (μ = 3.86 and 3.72 D, respectively) and relative permittivities (ϵ_*r*_ = 36.8 and 37.8 at 20 °C, respectively, Table 1) which lead to strong dipole–dipole interactions and relative orientational effects in the liquid structure.^12^ Both molecules are regarded as weak Lewis bases, with donor numbers (DN) of 26.6 kcal mol^–1^ and 27.8 kcal mol^–1^ and acceptor numbers (AN) of 16.0 kcal mol^–1^ and 13.6 kcal mol^–1^, respectively, for DMF and DMAc, Table 1.^13^ Furthermore, the presence of a C(=O)–H group in DMF raises the possibility of hydrogen bonding by a weakly donating formic H atom. On a practical level, this functionality also means that while DMF is one of the most heavily used solvents for chemical synthesis, it can react under highly basic conditions and with strong reducing and chlorinating agents. DMAc is usually more inert and so has complementary applications for example in the production of pharmaceuticals and polymers.^11^

**Table 1:**
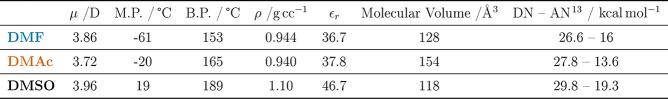
Selected Physicochemical Properties of the Liquids DMF, DMAc, and DMSO

Neutron diffraction studies of liquid DMF and DMAc have shown well-defined local structures.^12^ For both cases, the coordination number of molecules in the first solvation shell is found to be around 13, with a clear second shell also present. In DMF, weak C(=O)–H···O hydrogen bonds are observed that are thought to be electrostatic in nature. In DMF, the first solvation shell shows the expected preference for antiparallel dipole orientation between molecules, while in DMAc, parallel dipoles maximize dispersion forces between the π-delocalized O=C–N backbones and methyl groups.^12^ These results are consistent with Raman and IR spectroscopy and molecular dynamics studies of liquid DMF, all of which reported a highly structured first solvation shell with weak hydrogen bonding from carbonyl and methyl H atoms, in line with the “relatively short” C–H···O=C contacts seen earlier by gas-phase electron diffraction.^14−19^

Dimethyl sulfoxide (DMSO, Me_2_S=O) is a pyramidal molecule with a high dipole moment (μ = 3.96 D), weak Lewis base character (DN = 29.8 kcal mol^–1^ and AN = 19.3 kcal mol^–1^), and a remarkably high permittivity for an aprotic solvent (ϵ_*r*_ = 47.2 at 20 °C). Its unique properties are due to the combination of a soft lone pair on the sulfur atom and the strong polarization of the S=O bond. DMSO, like DMF and DMAc, is miscible with water and many organic solvents and has a unique ability to solvate a wide range of chemical species from apolar hydrocarbons to entirely dissociated salts. DMSO is therefore extremely important in processing and technology.^20^ Moreover, DMSO is able to penetrate human skin with a nondestructive effect on tissues and is a keystone protectant in cryobiology.^21^ Additional theoretical interest stems from the long-standing question of how to best represent the sulfoxide bond: S^+^–O^–^ rather than a formal S=O double bond.^22−24^ Structural studies of liquid DMSO by X-ray and neutron diffraction have reported nearest neighbor coordination numbers in the range 11.5–13.8 and provided evidence for short-range antiparallel alignment of dipoles, with head-to-tail ordering at longer distances. In addition, weak methyl hydrogen to oxygen intermolecular contacts were observed at distances of approximately 3.4 Å, but as such, these probably do not constitute hydrogen bonds.^25−28^

While the bulk physicochemical properties of DMF, DMAc, and DMSO are therefore similar (Table 1), the pure liquids exhibit contrasting local structures. This latter point immediately raises the question as to which interactions will dominate in mixtures of these molecules, and in particular how, and to what extent, DMSO is accommodated within the local solvation environments of DMF and DMAc (and *vice versa*).

Previous studies of mixtures of the polar protic solvent NMF in DMSO by neutron diffraction point to the formation of a strong N–H···O=S hydrogen bond between the protic amine group of NMF and the oxygen of DMSO at 1.6 Å.^29^ The latter distance is considerably shorter than the typical strong hydrogen bonding in the liquid state: taking the interaction between water molecules as an example, the first O–H···O contact is found at 1.85 Å at ambient conditions.^30,31^ NMF–DMSO hydrogen bonding is more similar in length to liquid HF, one of the shortest hydrogen bonds reported in a liquid.^32^ As a consequence, NMF and DMSO molecules form very stable dimers in the mixtures. This in turn results in well-organized first and second solvation shells with a preference for heteromolecular NMF–DMSO hydrogen bonds, rather than homomolecular NMF–NMF.^29,33−36^ NMF, though, is a protic, highly polar solvent (μ = 3.86 D), with extremely high relative permittivity (ϵ_*r*_ = 181 at 25 °C) and the ability to act as both proton donor and acceptor via its N–H and C=O groups. In clear contrast to NMF, DMF and DMAc are aprotic solvents that only form weak hydrogen bonds via the C–H and methyl groups. As such, they will pose a very different conundrum for DMSO as a cosolvent when they are compared with NMF.

In this study, we have used neutron diffraction in conjunction with isotopic substitution of hydrogen (H) by deuterium (D) to study both the pure liquid amides DMF and DMAc and equimolar 50:50 mixtures of these with DMSO to understand the role of weak intermolecular interactions, such as weak hydrogen bonding and dispersion forces, on a molecular level and to reveal the role of DMSO as the cosolvent in an aprotic environment. The use of the Empirical Potential Structure Refinement (EPSR) computations has allowed us to uncover the 3-dimensional site–site correlations in these systems.^37^

## Theoretical Basis

The function of interest which can be extracted from a neutron diffraction measurement is known as the total structure factor, *F*(*Q*), which can be written as
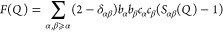
1where *c*_α_ and *c*_β_ and *b*_α_ and *b*_β_ are respectively the fractional concentrations of the atomic species α and β and the (isotope dependent) coherent neutron scattering lengths,  is the magnitude of the neutron scattering vector, and *S*_*αβ*_(*Q*) is the Faber-Ziman partial structure factor for any two types of atoms. There is, therefore, a unique *F*(*Q*) for each isotopic composition (isotopologue) of a sample. In particular, we can exploit the difference in sign and magnitude between the coherent neutron scattering lengths of hydrogen (*b*_*H*_ = −3.74 fm) and deuterium (*b*_*D*_ = 6.72 fm) to distinguish between specific sites in a molecule and thereby measure multiple distinct *F*(*Q*)s. This approach constrains the overall structure refinement so that we can interrogate the individual site–site correlations needed to describe the structure of a complex liquid.^38−40^

The partial structure factors are related to the partial radial distribution functions (RDFs), *g*_*αβ*_(*r*), via Fourier transformation
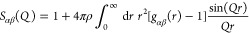
2where ρ is the atomic number density, and *r* is the distance between two species α and β. The *g*_*αβ*_(*r*)s represent the probability density of finding, by spherical averaging, an atom of species β at distance *r* from an atom of species α chosen as the origin of the reference system. These functions therefore contain important site-specific structural information on the sample.^40^ In a liquid system, *g*_*αβ*_(*r*) tends to 1 at large values of *r*.

In order to quantify the average coordination number, *N*_*αβ*_(*r*_0_), of sites of type β in proximity to a site of type α up to a maximum distance *r*_0_, one can integrate the partial radial distribution function *g*_*αβ*_(*r*) over the separation distance, *r*
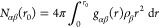
3where ρ_β_ is the number density of species β, and *r*_0_ is the maximum distance of integration. By definition, the first coordination number gives the average number of sites of species β present in a sphere of radius *r*_0_ centered on a site of species α. Traditionally, the upper limit of the integral in eq 3 is the position of the first minimum of the partial *g*_*αβ*_(*r*). Alternatively, the cumulative coordination numbers can be plotted as a function of the distance *r* from the central species.

Beyond a one-dimensional analysis, the Spatial Density Functions (SDFs) are a three-dimensional map of the density of neighboring molecules around a central molecule as a function of angular distance, *r*, and angular position, θ. The SDFs therefore represent regions of space around a central molecule that are most likely to be occupied by a molecule of the same or another species at a given distance.^41,42^

## Experimental Details

Experimental data have been acquired at the Near and InterMediate Range Order Diffractometer (NIMROD) at the ISIS Neutron and Muon Source (Didcot, UK) across a *Q* range of 0.05 Å^–1^–50 Å^–1^.^43^ This wide *Q* range provides high resolution in real space. DMF, DMAc, and DMSO and their isotopes were purchased from Sigma-Aldrich with purities ≥99.5% and handled under inert atmosphere. For pure DMF and DMAc, fully hydrogenated, fully deuterated, and a 50:50 mixture of hydrogenated and deuterated liquids were loaded into Ti_0.68_Zr_0.32_ null scattering cells to give a total of 3 isotopically distinct samples for each liquid amide. To produce 50:50 amide/DMSO mixtures, the anhydrous liquids were mixed to obtain 7 isotopically distinct samples for DMF/DMSO and 5 for DMAc/DMSO, as summarized in Table 2. The samples were inserted into flat-plate null coherent scattering titanium/zirconium cells, with a 1-mm sample and wall thicknesses. Each composition was run for a minimum of 2 h at 298 K. Data for the pure liquid DMF and DMAc have been reanalyzed using the same methods and protocols as the mixture to allow for a more rigorous comparison.^12^ To allow data correction and calibration, scattering data were also collected from the empty instrument, empty sample cells, and an incoherent scattering vanadium–niobium reference slab of thickness 3 mm. Reduction of the experimental data, including absolute normalization, background subtraction, and multiple and inelastic scattering corrections, has been conducted using standard procedures as implemented within the Gudrun package.^44^

**Table 2:**
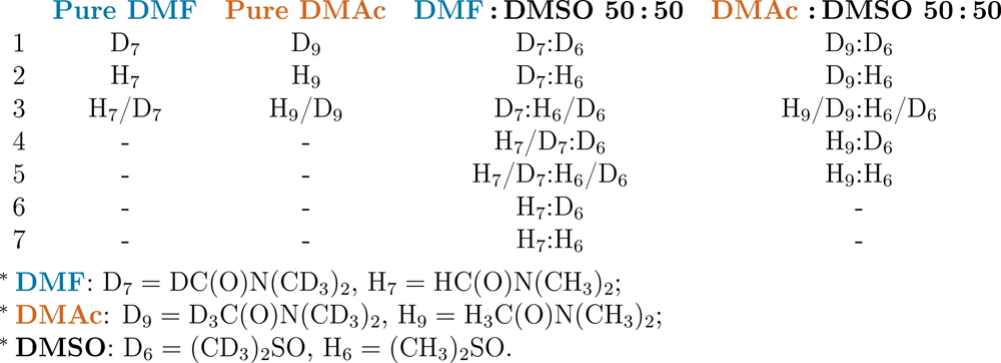
List of the Isotopically Distinct Samples Run in the Neutron Experiments^a^

a The asterisks in the table denote DMF, DMAc, and DMSO isotopologues.

## Computational Details

The EPSR method consists of a classical Monte Carlo molecular simulation which takes initial seed potentials for modeling pairwise interactions and subsequently refines these through the incorporation of an empirical potential. This empirical potential is calculated with reference to any mismatch between the experimental and simulated data, until a satisfactory agreement between the calculated structure factors and the measured neutron scattering data is reached. In this manner, a three-dimensional structural model of the system can be obtained which is consistent with the experimental data.

**Figure 2:**
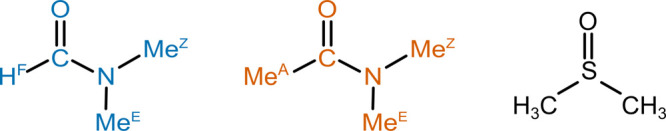
DMF, DMAc, and DMSO molecular models with relevant atomic sites labeled.

The intermolecular potential between two atomic sites α and β is modeled in EPSR via a Lennard–Jones 12-6 function plus a Coulombic term
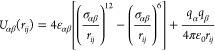
4where *q*_α,β_ is the atomic partial charges, and ϵ_*αβ*_ and σ_*αβ*_ are the well depth parameter and the range parameter, respectively, and are given by the Lorentz–Berthelot mixing rules in terms of their values of the individual atoms.^45^ All the molecules were generated in *Avogadro*, and their geometry is optimized for 500 steps using the MMFF94 force field^46,47^ (see Table S1 for intramolecular parameters). The intermolecular seed potentials are taken from the OPLS series of force fields (see Table 3).^48−51^ The cubic EPSR boxes of side lengths 44.80 and 47.58 Å contain 700 molecules for pure liquid DMF and DMAc, while cubic boxes of side lengths 49.77 and 51.63 Å contain 1000 molecules of which 500 are DMF/DMAc and 500 are DMSO. The atomic number densities for the four systems are 0.0934 atoms/Å^3^ and 0.0975 atoms/Å^3^ for the DMF and DMAc pure liquid and 0.08925 atoms/Å^3^ and 0.09080 atoms/Å^3^ for DMF/DMSO and DMAc/DMSO mixtures. These values are obtained from a weighted average of the relevant bulk densities of the pure liquids and verified by reference to the overall scattering levels of the experimental data. The labels assigned to the atomic sites of the DMF, DMAc, and DMSO molecules are shown for clarity in Figure 2.

**Table 3:**
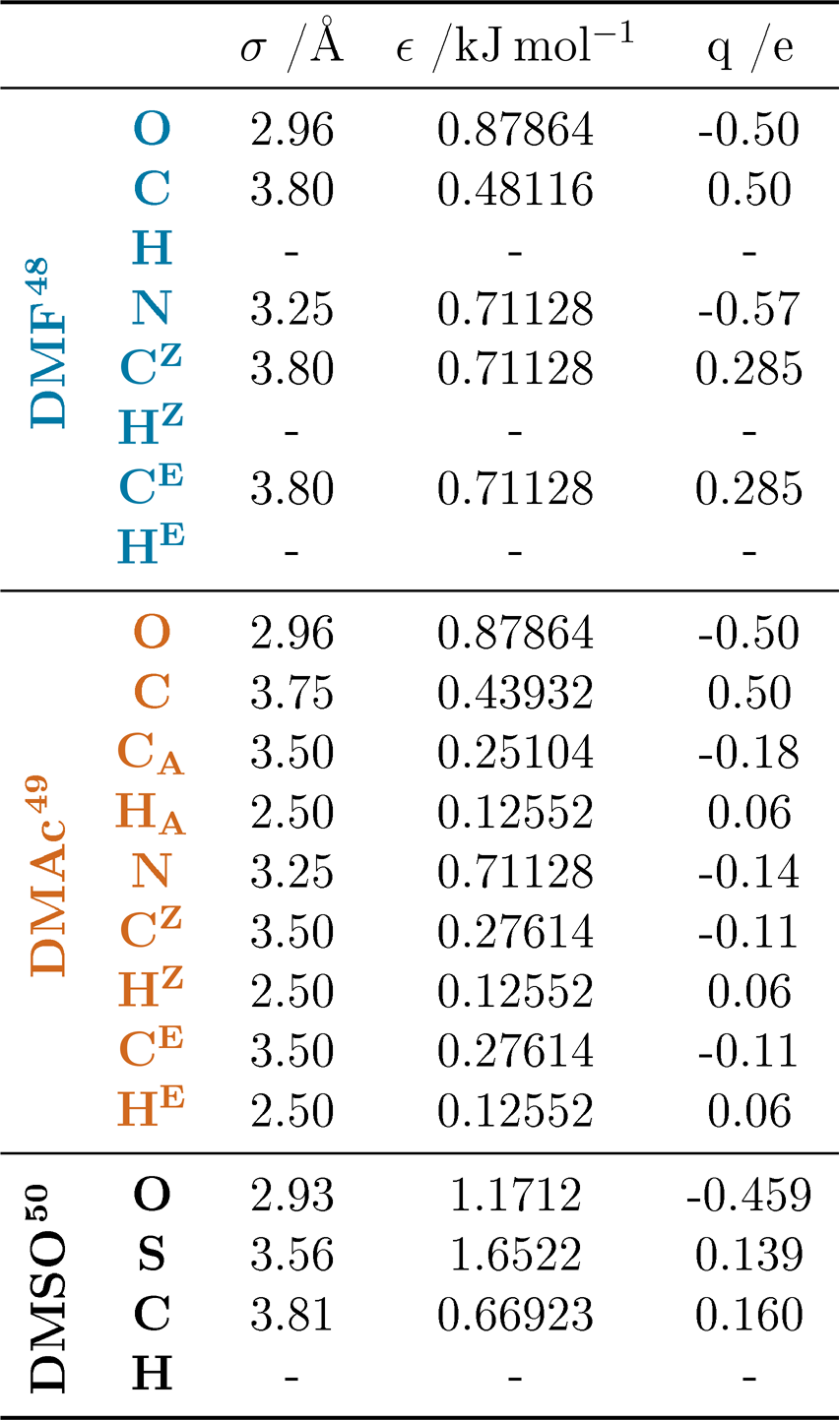
Lennard-Jones Parameters and Charges for DMF,^48^ DMAc,^49^ and DMSO^50,51^ from Top to Bottom^a^

a Atomic sites correspond to the labeling of Figure 2.

## Results and Discussion

**Figure 3:**
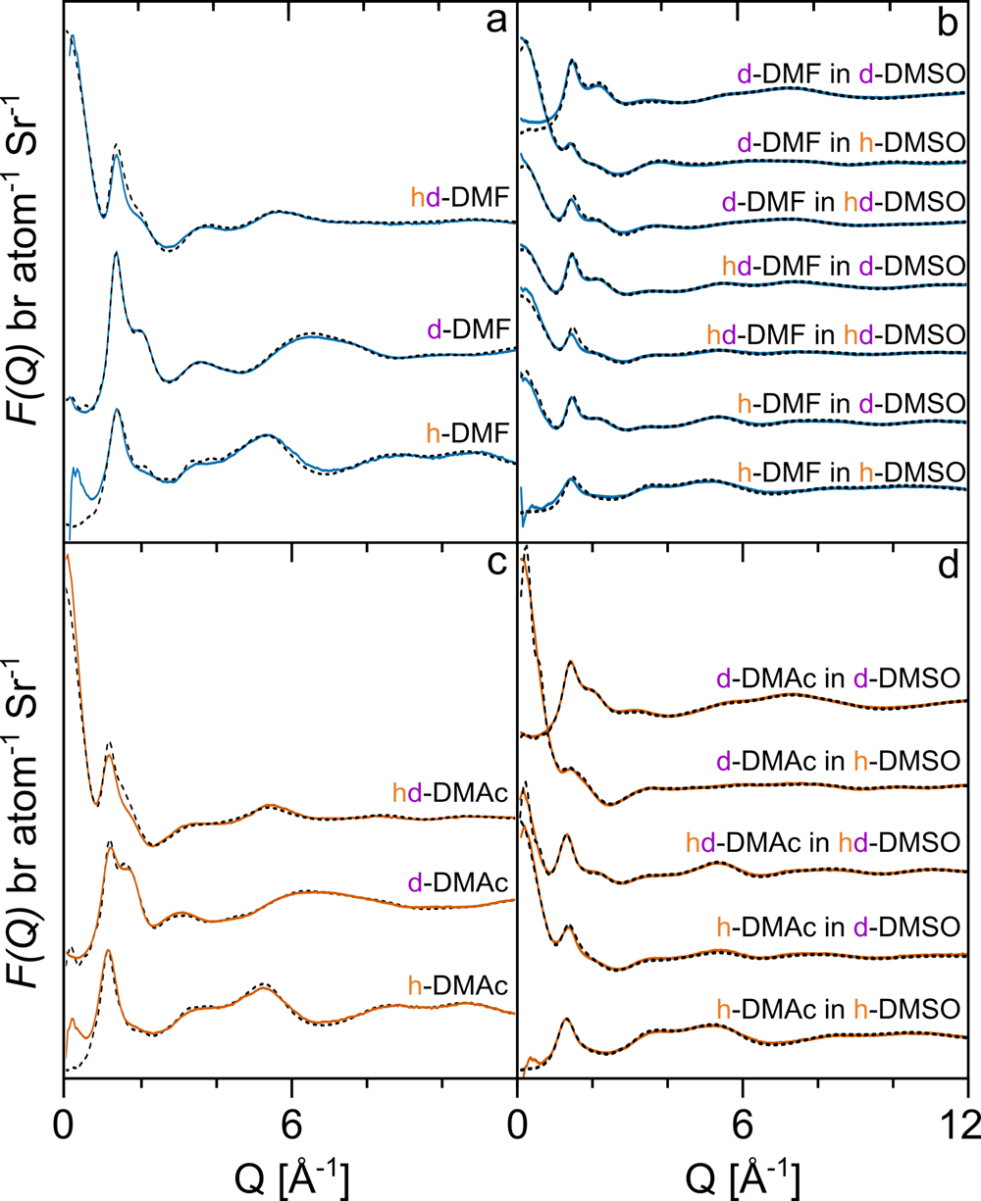
Experimental (solid line) and modeled (dashed line) neutron diffraction total structure factors, *F*(*Q*), for pure liquid DMF (a) and the DMF/DMSO (b) mixture (left) and pure liquid DMAc (c) and the DMAc/DMSO (d) mixture (right).

The isotopically distinct experimental neutron diffraction structure factors, *F*(*Q*), are plotted for the pure liquid amides and their DMSO mixtures against the EPSR model in Figure 3. Excellent agreement between the experimental data and model has been achieved for each data set; the small discrepancies at low-*Q* in fully hydrogenated samples such as h-DMF and h-DMAc are attributed to a residual presence of inelastic and multiple scattering events.^44,52^ The rise in low-*Q* scattering for samples such as hd-DMF and hd-DMAc can then be attributed to the fact that these liquids are comprised of a mixture of fully hydrogenated and fully deuterated molecules, Table 2. This isotopic partitioning on individual molecules leads to a genuine rise in elastic scattering that is well captured by the EPSR model. These neutron diffraction data (Figure 3 solid line) show clearly that the liquid amides are fully miscible with DMSO at this concentration, as there is an absence of any residual low-*Q* signal that would indicate the presence of homomolecular clustering. This observation can be confirmed by examining the molecular center-of-mass (CoM) radial distribution functions, *g*_*CoM*–*CoM*_(*r*), obtained from the EPSR model.

**Figure 4:**
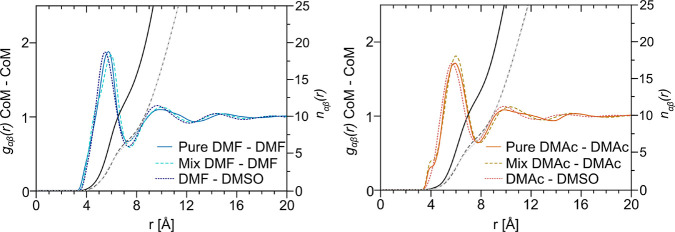
CoM–CoM intermolecular partial radial distribution functions, *g*_*CoM*–*CoM*_(*r*), and cumulative coordination numbers, *N*_*CoM*–*CoM*_(*r*), for DMF (left) and DMAc (right). Note that the RDFs for the pure liquids and the mixtures are very similar, showing that DMSO does not disrupt the amide–amide correlations as it infiltrates the local coordination. In addition, we observe clear second and third solvation shells which, if anything, become more ordered in the presence of DMSO.

Figure 4 and Table 4 present the radial distribution functions (RDFs) for the CoM amide–amide interactions, *g*_*CoM*–*CoM*_(*r*), and the cumulative coordination number, *N*_*CoM*–*CoM*_(*r*), as a function of the separation distance *r*. These RDFs generated by EPSR are compared with those obtained by classical Monte Carlo simulation in Supporting Information Section S5. We see immediately from these functions that the local structure of liquid DMF and DMAc, as depicted by the CoM–CoM RDFs, is almost unaltered by the presence of DMSO in the mixtures. Moreover, the plotted and tabulated cumulative coordination numbers confirm that approximately half of the ∼13–14 first shell amide molecules in the pure liquids are replaced by DMSO in the mixtures. This corresponds to near ideal coordination shell mixing at the molecular level. In addition, the RDFs provide clear evidence for a second and third solvation shell.

**Table 4:**
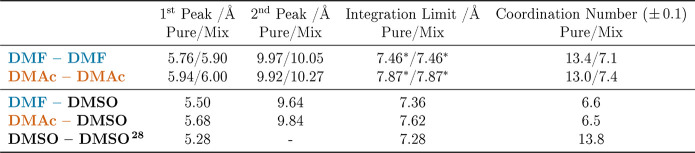
CoM–CoM First and Second Peak Positions, Integration Limits, and Coordination Numbers in the Pure Liquid Amides and the 50:50 Mixtures with DMSO^a^

a The asterisks in the table denote the following: the integration limit is set to the first minimum of the mix to allow more direct comparison between the corresponding coordination numbers.

Our CoM–CoM RDF data do reveal subtle structural differences in peak features and positions, for example a small shortening of the heteromolecular amide–DMSO first peak relative to that of the amide–amide (Table 4) and concomitant slightly enhanced definition of the second and third peaks in the mixtures. In both systems, the first peak of the CoM–CoM RDF, associated with the homomolecular first solvation shell of DMF (Figure 4, left) and DMAc (Figure 4, right), shifts to marginally longer distances in the presence of DMSO. However, when interpreting these rather nuanced effects, we must bear in mind that the CoM for each molecule (Figure 1) is in a different position relative to the C=O group and that they possess different, albeit comparable, molecular volumes (Table 1). The *N*_*CoM*–*CoM*_(*r*) for the pure liquids shows that at the distance of the first minimum of *g*_*CoM*–*CoM*_(*r*) DMF and DMAc are surrounded by an average of 13.4 and 13.0 neighbors, respectively. When in a 50:50 mixture, the composite coordination shells are made up of ∼7 amide molecules and ∼6.5 DMSO, giving total coordination numbers of 13.7 and 13.9 for DMF and DMAc, respectively. Our data therefore indicate that the total coordination shells in the mixtures are remarkably similar to those in the pure amides and that DMF and DMAc are almost equally solvated by other molecules of the same species as by DMSO. This contrasts with NMF/DMSO mixtures, where there was a strong preference for NMF–DMSO contacts due to N–H···O hydrogen bonding.^29^

**Figure 5:**
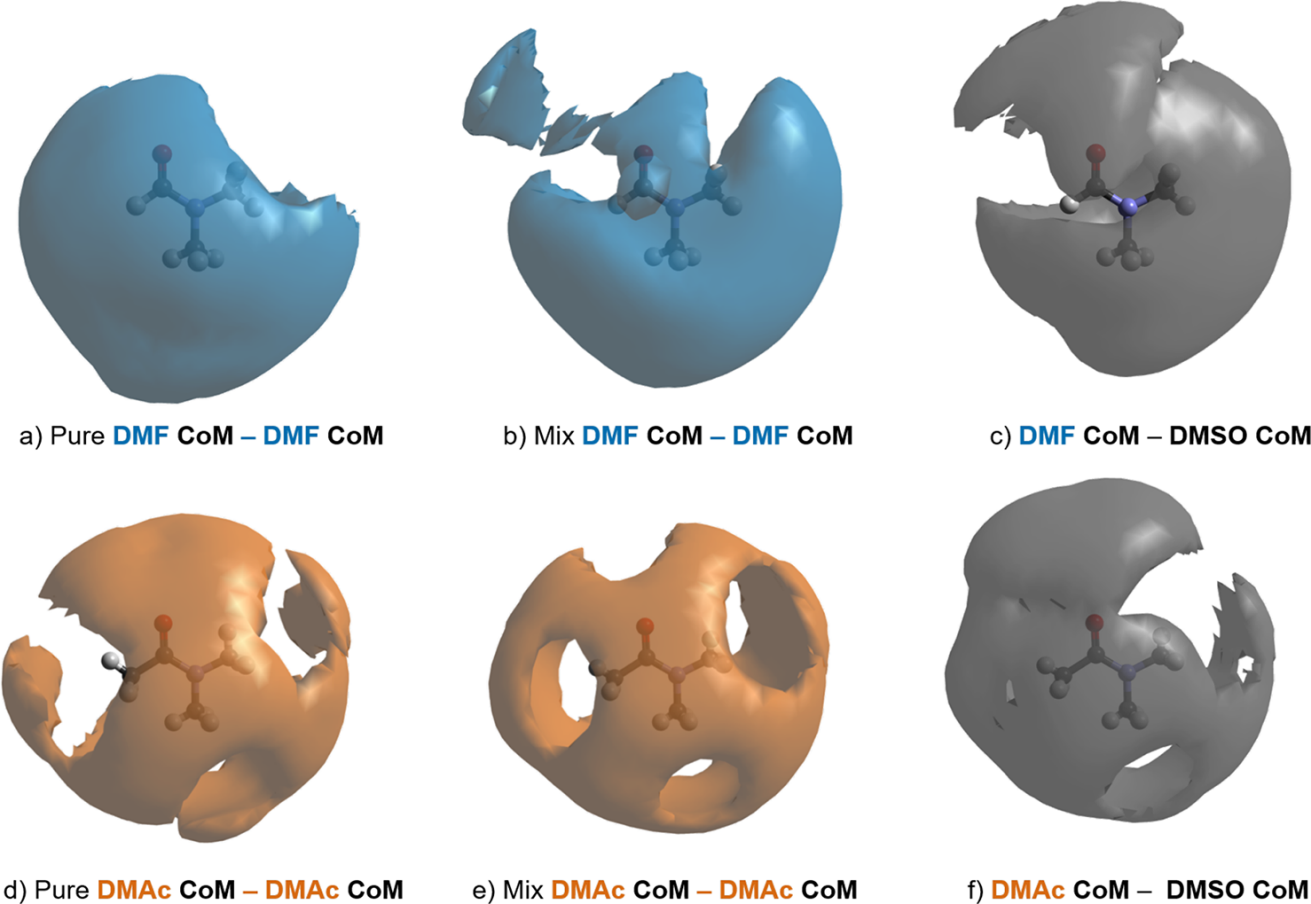
CoM–CoM spatial density functions (SDFs) representing the 25% most likely configuration of (a,b) the DMF molecule CoM around another DMF up to 7.5 Å from the DMF center-of-mass (CoM) in bulk liquid amide (left) and in the mixture with DMSO (right); (d,e) the DMAc molecule CoM around another DMAc up to 7.9 Å from the DMAc CoM in bulk liquid amide (left) and in the mixture with DMSO (right); (c,f) the DMSO molecule CoM around a DMF and a DMAc up to 7.5 and 7.9 Å distances from the DMF and DMAc CoM, respectively.

The CoM–CoM RDFs and cumulative coordination numbers reflect the radially averaged packing structure of the solutions. By interrogating the EPSR model, we are also able to extract the 3-dimensional CoM–CoM spatial density functions (SDFs). These typically reveal whether there is any directional preference within the local coordination environments. With this in mind, Figure 5 presents the CoM–CoM SDFs for the pure liquids and mixtures. These functions show that the first coordination shell is distributed broadly over the molecular spheroid, particularly over the C=O groups and the O=C–N backbone. As one might expect, lacunae occur over the methyl groups (H^A^, H^Z^, and H^E^). However, we see no clear preference for DMSO over DMF/DMAc, except in the region of the C=O groups. We will examine this effect in detail by analyzing the atomic site specific RDFs, coordination numbers, and SDFs.

**Figure 6:**
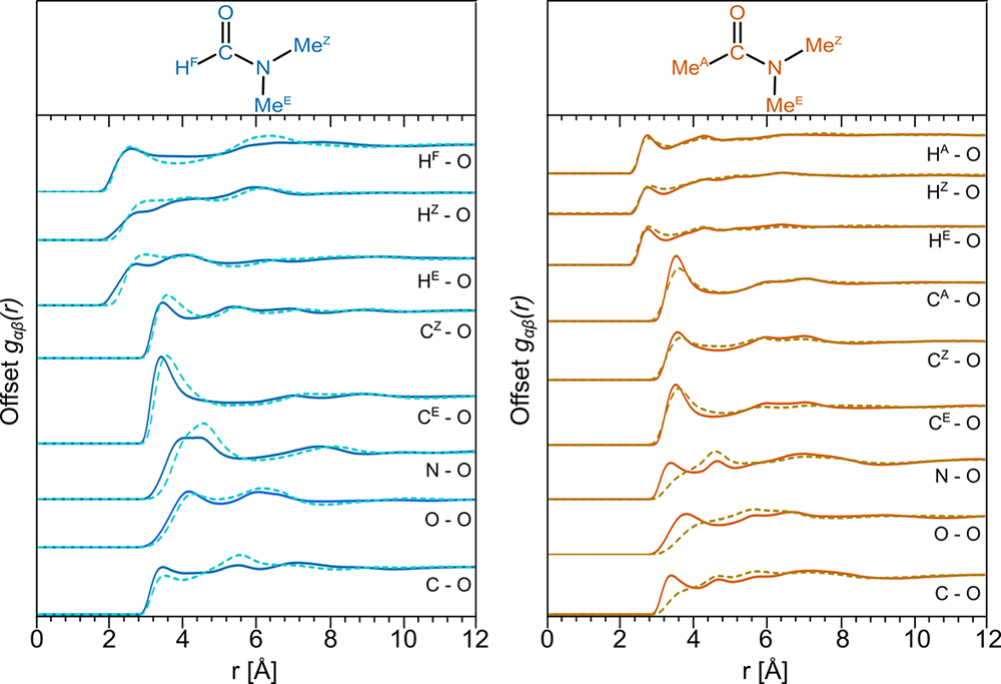
Amide–amide intermolecular partial radial distribution functions, *g*_*αβ*_(*r*)s, for DMF (left) and DMAc (right): pure liquids (solid lines) compared to the structure of DMF and DMAc in the equimolar mixtures with DMSO (dashed lines). Note the remarkable similarity between the pure and mixture functions for DMF–DMF and the slight outward shift on mixing for the N–O, O–O, and C–O distributions in the DMAc system.

To understand the intermolecular amide–amide interactions between two specific atomic sites, we can extract the relevant partial radial distribution, *g*_*αβ*_(*r*). Figure 6 reports selected *g*_*αβ*_(*r*)*s* for DMF (left) and DMAc (right) in both the pure liquid (solid line) and the mixture with DMSO (dashed line). The corresponding intermolecular partial structure factors, *S*_*αβ*_(*Q*), are presented in Supporting Information Figure S6. Table 5 provides the peak positions and coordination numbers, along with the integration limits. Note that in Table 5, we have used the same integration limit in the pure and mixed systems for the H–O correlations. Further pairs are given in Supporting Information Figures S3 and S4.

**Table 5:**
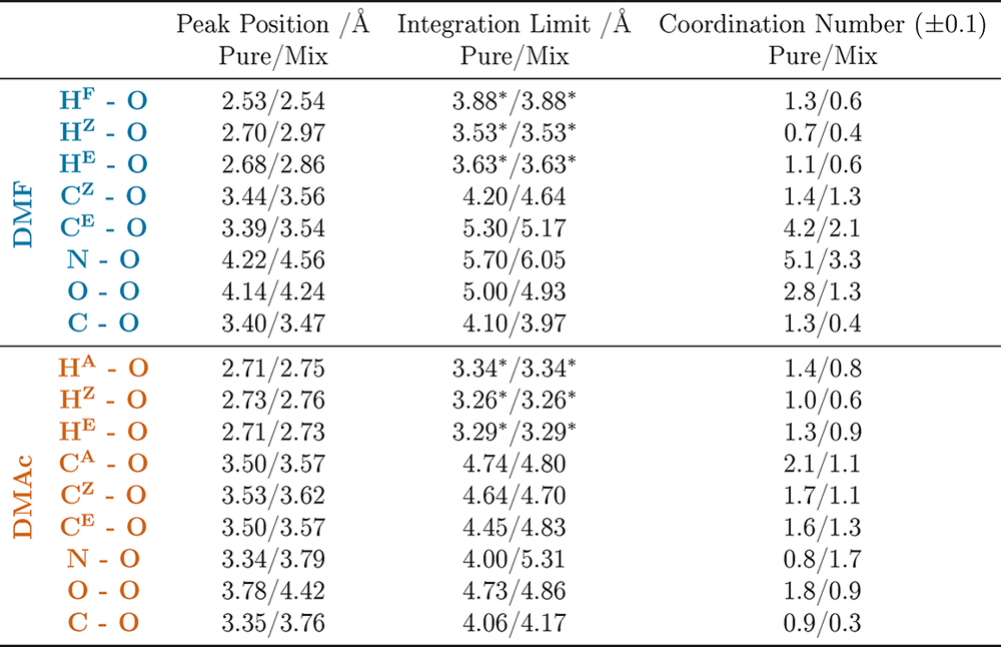
Amide–Amide Peak Positions, Integration Limits, and Coordination Numbers for Interactions between the Main Sites of Interest for DMF and DMAc in the Pure Liquids and the 50:50 Mixtures with DMSO^a^

a The asterisks in the table denote the following: the integration limit is set to the first minimum of the mix to allow more direct comparison between the corresponding H-bonding coordination numbers.

First, we focus on the amide–amide interactions between the various protons and the O(=C) site, *g*_*H*–*O*_(*r*). In DMF, the H^F^-to-oxygen distribution shows a peak at ∼2.5 Å, which is consistent with a weak hydrogen bond of electrostatic nature.^53^ The coordination numbers for this pair of sites in the pure and mixed systems are 1.3 and 0.6, respectively, in line with the overall mole fraction of DMF and indicative of no preference for/against DMSO. In both pure DMF and DMAc, the methyl protons H^A^, H^Z^, and H^E^ show maxima in the RDF at around 2.7 Å. This again is indicative of weak hydrogen bonding. Changes to the RDFs on mixing are subtle, but in the case of DMF, we observe peak shifts to ∼2.9 Å (H^Z^) and ∼3.0 Å (H^E^) in the presence of DMSO.

Turning now to the amide–amide O=C–N backbone correlations, in Figure 6, we plot the N–O, O–O, and C–O RDFs for both the pure and mixed DMF and DMAc systems. In the case of DMF–DMF, we observe N–O and O–O correlations with peak positions in the range 4.1–4.6 Å, with relatively mild perturbations when comparing the RDFs for the pure and mixed systems. As a general point, however, we note that such discrepancies between specific site–site correlations are not captured by the CoM-CoM RDF shown in Figure 4. The DMF–DMF *g*_*C*–*O*_(*r*)s have a first peak at ∼3.4 Å, consistent with the observed H(−C)···O hydrogen bond. From Figure 6 and Table 5, we see that nearest neighbor DMAc–DMAc backbone interactions have peak positions in the range of 3.3–3.8 Å, slightly shorter than their DMF–DMF counterparts. This confirms the importance of dispersion forces and the steric hindrance of the acetic methyl in pure DMAc.^12^ In contrast to the case of DMF, DMAc–DMAc backbone interactions are significantly displaced to longer distances in the presence of DMSO. We can investigate the origins of this effect by turning to the heteromolecular amide–DMSO interactions.

**Figure 7:**
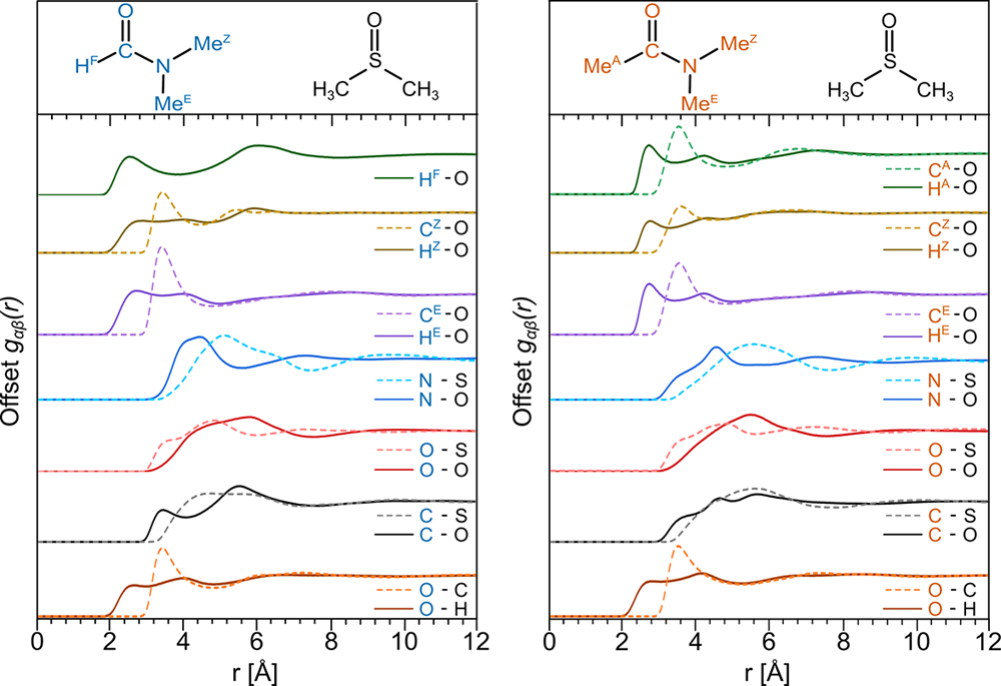
Amide–DMSO intermolecular partial radial distribution functions, *g*_*αβ*_(*r*)s, for DMF/DMSO (left) and DMAc/DMSO (right). Note that the approaches between DMF and DMAc with DMSO are very similar, and the main differences lie in the shorter interatomic distances in the case of DMF and are linked to the ability of the formic proton H^F^ to interact via C–H···O hydrogen bonding.

Selected amide–DMSO *g*_*αβ*_(*r*) partial RDFs are shown in Figure 7 for DMF/DMSO (left) and DMAc/DMSO (right), respectively, along with the corresponding peak positions and coordination numbers in Table 6. Further pairs are given in Supporting Information Figure S5.

**Table 6:**
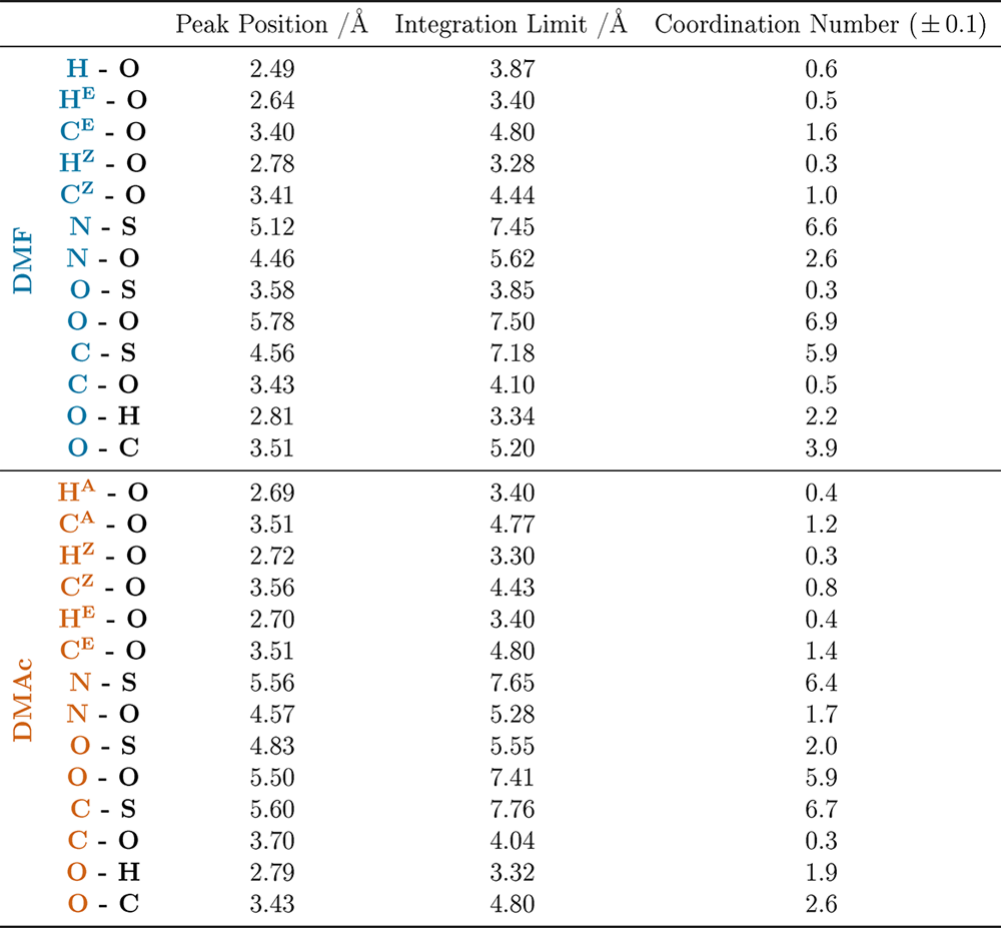
Amide–DMSO Peak Positions, Integration Limits, and Coordination Numbers with Reference to the Main Sites of Interest on DMF, DMAc, and DMSO

Regarding the heteromolecular hydrogen bonding, we note that for DMF, the formic proton H^F^ to DMSO oxygen distribution shows a peak at ∼2.5 Å. This is an indication of a weak electrostatic hydrogen bond, with the C(=O)–H···O=S distance almost identical to that observed for C(=O)–H···O=C between two DMF molecules (Table 5). Weak hydrogen bonds are also observed between amide methyl protons (H^A^, H^Z^ and H^E^) and DMSO oxygen and DMSO methyl protons and amide oxygen, at distances between ∼2.6 and 2.8 Å. This methyl C–H···O interaction is extremely weak for the sp^3^ carbon^54^ and in this case is facilitated by the high molecular dipoles of DMF, DMAc, and DMSO. The partial RDFs relative to the amide O=C–N backbone and the O, S, and C sites of DMSO are presented in Figure 7, with corresponding peak positions and coordination numbers in Table 6. In the case of DMF–DMSO, the N–O, O–O, C–O, and O–C partial RDFs show only subtle differences when compared with their amide–amide counterparts. This is consistent with our observation that DMF–DMF backbone RDFs are very similar in the bulk and mixed liquids. We attribute this relative insensitivity to the presence of a formic proton, H^F^, and consequent C(=O)–H···O hydrogen bond as a dominant structural motif in DMF–DMSO mixtures. This C(=O)–H···O interaction is absent in DMAc, where we observe only very weak Me–H···O hydrogen bonds and dispersion interactions. In this case, DMAc–DMAc backbone interactions were displaced to longer distances in the mixture. We see faint indications in the DMAc–DMSO RDFs that DMSO may compensate for this effect. Specifically, we point to the lower separation shoulders in *g*_*N*–*O*_(*r*) and *g*_*C*–*O*_(*r*) occurring at around 3.4 Å. To obtain more detailed insight into the solvation shells, we need to look beyond the radially averaged representations of *g*_*αβ*_(*r*) and *N*_*αβ*_(*r*), and we therefore turn again to the 3-dimensional spatial density functions (SDFs).

**Figure 8:**
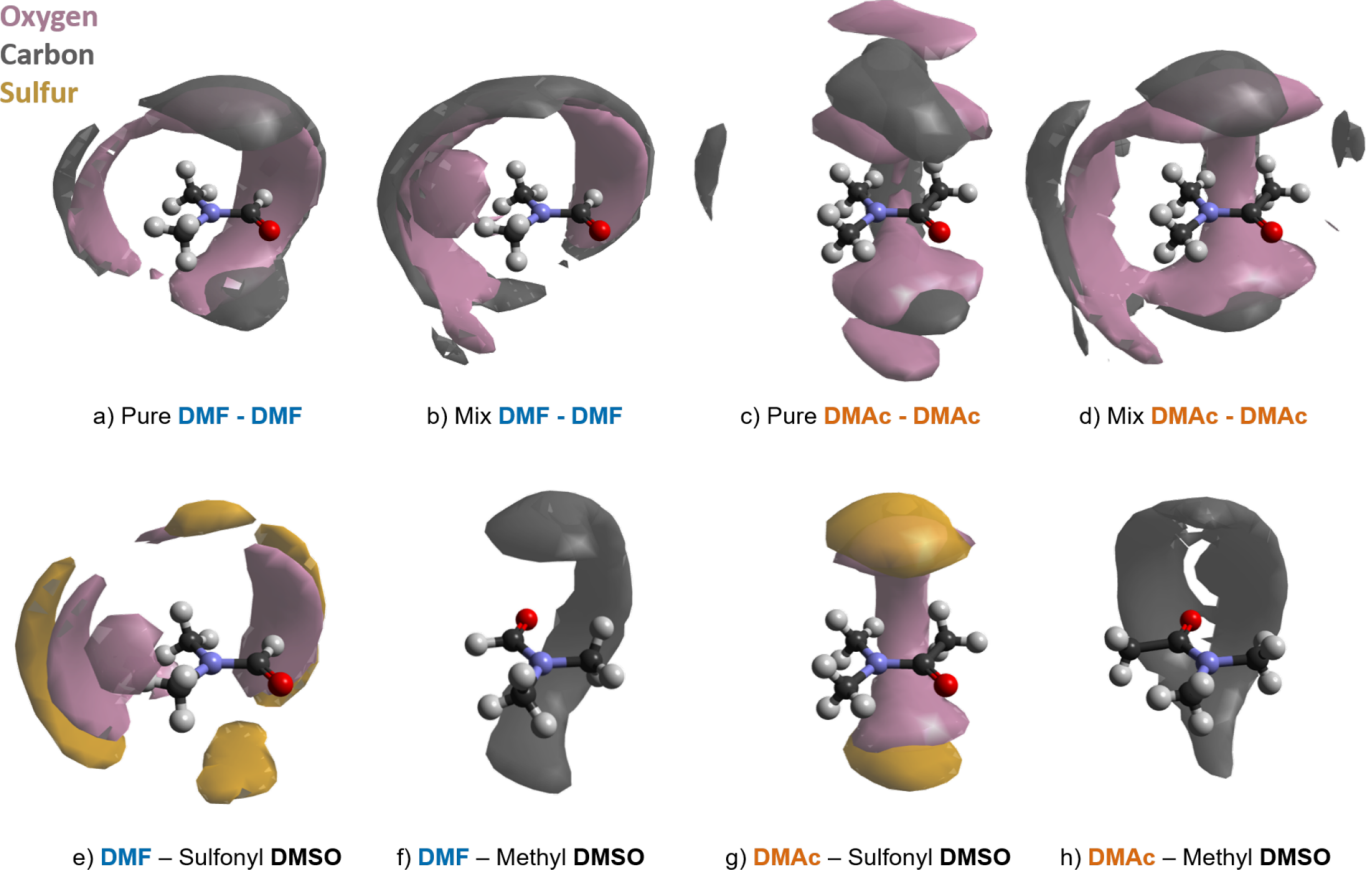
Amide–amide and amide–DMSO spatial density functions (SDFs) representing the following: (a,b) the 7% most likely configuration of the DMF C(black)=O(pink) group up to 7.5 Å from the DMF CoM in bulk liquid amide (left) and in the mixture with DMSO (right); (c,d) the 7% most likely configuration of the DMAc C(black)=O(pink) group up to 7.9 Å from the DMAc CoM in bulk liquid amide (left) and in the mixture with DMSO (right); (e,f) the 5% most likely position of the DMSO S(orange)=O(pink) group up to 7.35 and 7.6 Å distances from the DMF and DMAc CoM, respectively; (g,h) the 5% most likely position of the DMSO methyl carbons (black) up to 7.35 and 7.6 Å distances from the DMF and DMAc CoM, respectively. Note that panels (f,h) have been rotated for clarity.

Site-specific spatial density functions are displayed for pure amides and amide–DMSO mixtures in Figure 8. In each case, the reference molecule is shown at the CoM origin, and we focus on the 3-dimensional distribution of neighboring C=O and S=O groups. In these SDFs, carbonyl carbon density is shown in dark gray, oxygen in pink, and sulfur in orange.

The DMF–DMF functions (Figure 8, panels a and b) show a band of oxygen density, with more distant carbonyl carbon, centered around the formic proton. This feature extends at longer distances toward the methyl Me^Z^ groups and, through a narrow strip, toward Me^E^ and Me^Z^. These features confirm the presence of C(=O)–H···O and more distant Me–H···O hydrogen bonds. Overall, the pure and mixed DMF systems have broadly similar amide–amide spatial density, as predicted from our analysis of the RDFs. Relatively delicate changes to the DMF SDFs occur on mixing and are centered on the lobes corresponding to weaker interactions to the methyl groups, away from the formic proton. The DMF–DMSO SDFs (Figure 8, panels e and f) support our asseveration that the local solvation environment is occupied by DMF and DMSO in equal manner in the mixture. We note that the sulfonyl oxygen density tracks the main features of the amide oxygen, and, likewise, the sulfonyl sulfur and carbonyl carbon. The most likely location for DMSO methyl carbons is a band centered around the amide oxygen, indicative again of weak, directionally rather unconstrained, Me–H···O hydrogen bonds.

The DMAc–DMAc functions (Figure 8, panels c and d) show clearly contrasting behavior to those of DMF. In the pure liquid, the most likely DMAc oxygen and carbonyl carbon density is symmetrically above and below the plane of the O=C–N backbone, with a connecting strip directed toward the methyl protons H^A^ and H^E^. This picture is fundamentally different than that for DMF shown in Figure 8 panels a and b. Also in contrast to DMF, for DMAc there are clear differences in the amide–amide SDFs for the pure and mixed systems. Specifically, Figure 8 panel d shows an additional lobe directed toward the methyl protons H^Z^ and H^E^ and concomitant loss of density in the features seen in the pure system, panel c. The reason for this behavior becomes clear when we examine the DMAc–DMSO SDFs. We see that in the mixture, DMSO sulfonyl oxygen and sulfur mimic the closest approach of DMAc carbonyl oxygen and carbon in the pure liquid. We conclude that DMSO prefers to approach DMAc from above and below the plane of the O=C–N backbone or axially around Me^A^ and Me^E^. In doing this, DMSO molecules displace some of the DMAc–DMAc interactions observed in the pure liquid, thereby displacing the DMAc–DMAc density in the mixture toward Me^Z^ and Me^E^. This is entirely consistent with the shifts observed in the DMAc–DMAc backbone RDFs (Figure 6). As with DMF, the most likely location for DMSO methyl carbons is a band centered around the amide oxygen but broadened and cleft due to the presence of Me^A^.

## Conclusions

Neutron diffraction augmented by isotopic substitution of hydrogen (H) for deuterium (D) has been used to study pure DMF and DMAc and equimolar mixtures of DMF in DMSO and DMAc in DMSO in the liquid state. The atomistic sensitivity provided by neutron diffraction is critical for understanding weak intermolecular interactions on a molecular level, as these bonding motifs are elusive and often invisible to many experimental techniques. Empirical Potential Structure Refinement (EPSR) has been used to generate 3-dimensional atomistic models that are consistent with the experimental data. This approach has enabled us to uncover individual site–site interactions and to conduct a detailed comparison between the pure and mixed systems. Our scattering data show that the amides and DMSO are mixed on the nanoscale. Analysis of the EPSR center-of-mass (CoM) correlations shows that in all of our systems the coordination shell contains ∼13–14 molecules and that in the mixed systems there is, on average, near ideal solvation shell sharing between amide and DMSO. Examination of site-specific correlations reveals a rich structural landscape, in which replacement of the formic proton H^F^ in DMF by the methyl group Me^A^ in DMAc leads to fundamentally different solvation environments for these amides in both the pure liquids and mixtures. Weak C–H···O hydrogen bonds are formed by formic (DMF) and methyl (DMF, DMAc) protons, with distances around 2.5 and 3.0 Å. In addition, we observe dispersion interactions above and below the plane of the O=C–N amide π–backbones, particularly in the DMAc systems in which dispersive forces are expected to be predominant. By comparing pure amides with the liquid mixtures, we show that DMSO has a noteworthy ability to share in hydrogen bonding to both formic and methyl groups, matching the bond distances observed in the pure amides leading to similar solvation motifs and perfect mixing. As a result, DMSO is able to penetrate the amide–amide solvation shells while causing only subtle disruption to the amide–amide interactions. The new knowledge provided by our study is particularly important to tailor electrolytes in confined geometries such as battery electrodes and supercapacitors, since molecular mixing and local interactions are likely to impact on performance.

## Supporting Information Available

Total radial distribution functions, molecular bond lengths and angles, additional partial radial distribution functions, intermolecular partial structure factors, and classical Monte Carlo simulations (PDF)
