# Supplementary material for: Weak Interactions in Dimethyl Sulfoxide (DMSO)–Tertiary Amide Solutions: The Versatility of DMSO as a Solvent
Source: J Phys Chem B. 2023 Feb 8;127(6):1357–66. doi: 10.1021/acs.jpcb.2c07155 (PMC9940205; doi:10.1021/acs.jpcb.2c07155)
Supplement: Supplementary file 1 — jp2c07155_si_001.pdf [file jp2c07155_si_001.pdf]

Supporting Information
Weak Interactions in Dimethyl Sulfoxide (DMSO) – Tertiary Amide Solutions: the Versatility of DMSO as a Solvent

Camilla Di Mino,† Adam J. Clancy,‡ Andrea Sella,‡ Christopher A. Howard,†
Thomas F. Headen,¶ Andrew G. Seel,*,¶ and Neal T. Skipper*,†

†*Department of Physics and Astronomy, University College London, Gower Street, London WC1E 6BT, UK*

‡*Department of Chemistry, University College London, 20 Gordon Street, London WC1H 0AJ, UK*

¶*ISIS Neutron and Muon Source, Science and Technology Facilities Council, Rutherford Appleton Laboratory, Harwell Campus, Didcot OX11 0QX, UK*

E-mail: andrew.seel@stfc.ac.uk; n.skipper@ucl.ac.uk

Phone: +44 (0)1793 547500; +44 (0)207 679 3526

S1: Total Radial Distribution Functions *G(r)*

The total radial distribution function $G(r)$ is the weighted sum of the partial distribution functions $g_{\alpha\beta}(r)$ and related to the structure factor $F(Q)$ by the Fourier transform:[1]

$$G(r) = \sum_{\alpha,\beta\geqslant\alpha} (2-\delta_{\alpha\beta})\, b_\alpha b_\beta c_\alpha c_\beta \,(g_{\alpha\beta}(r)-1). \tag{S1}$$

The *G(r)s* of the pure liquid amides are shown in Figure S1, while the DMF/DMSO and DMAc/DMSO in Figure S2.

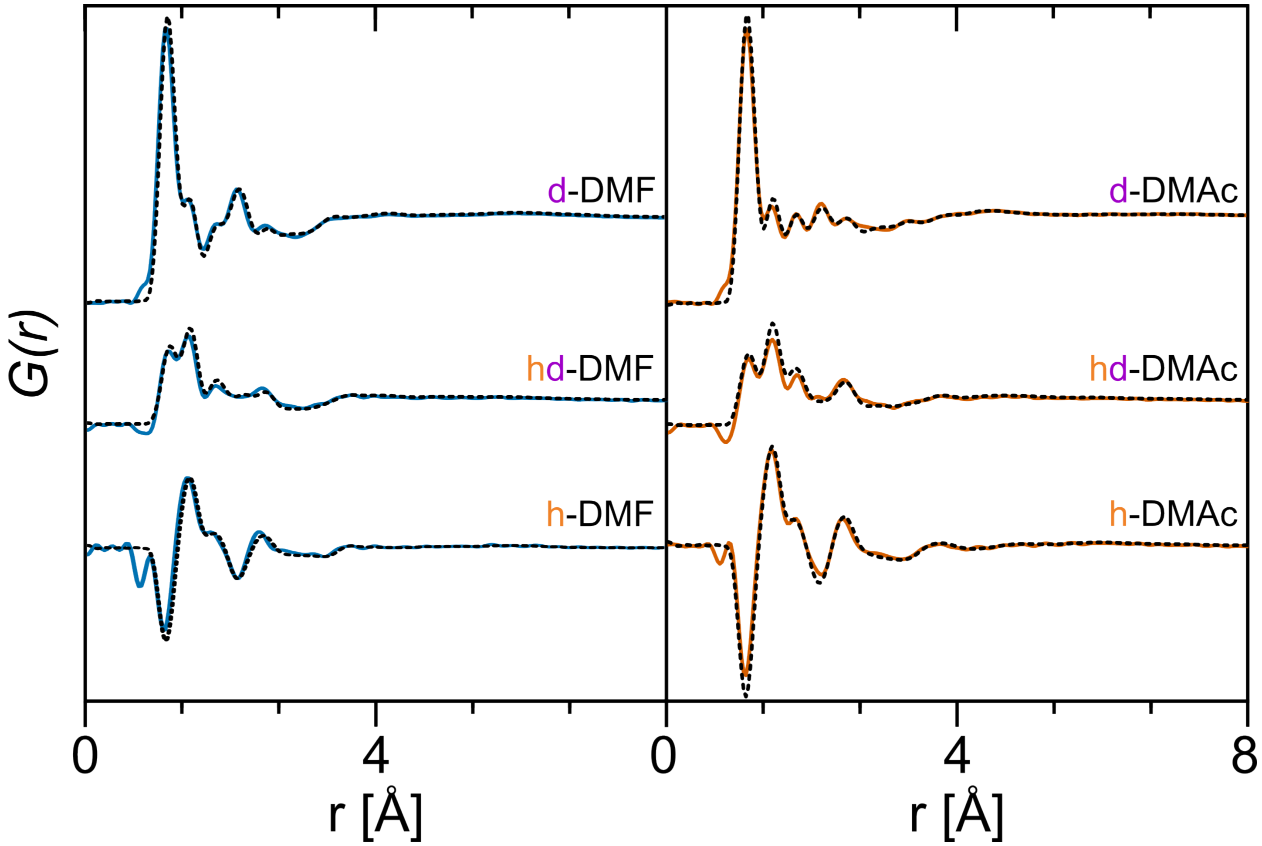

Figure S1: Experimental (solid–line) and modelled (dashed–line) neutron diffraction total radial distribution functions, *G(r)*, for pure liquid DMF (left) and pure liquid DMAc (right).

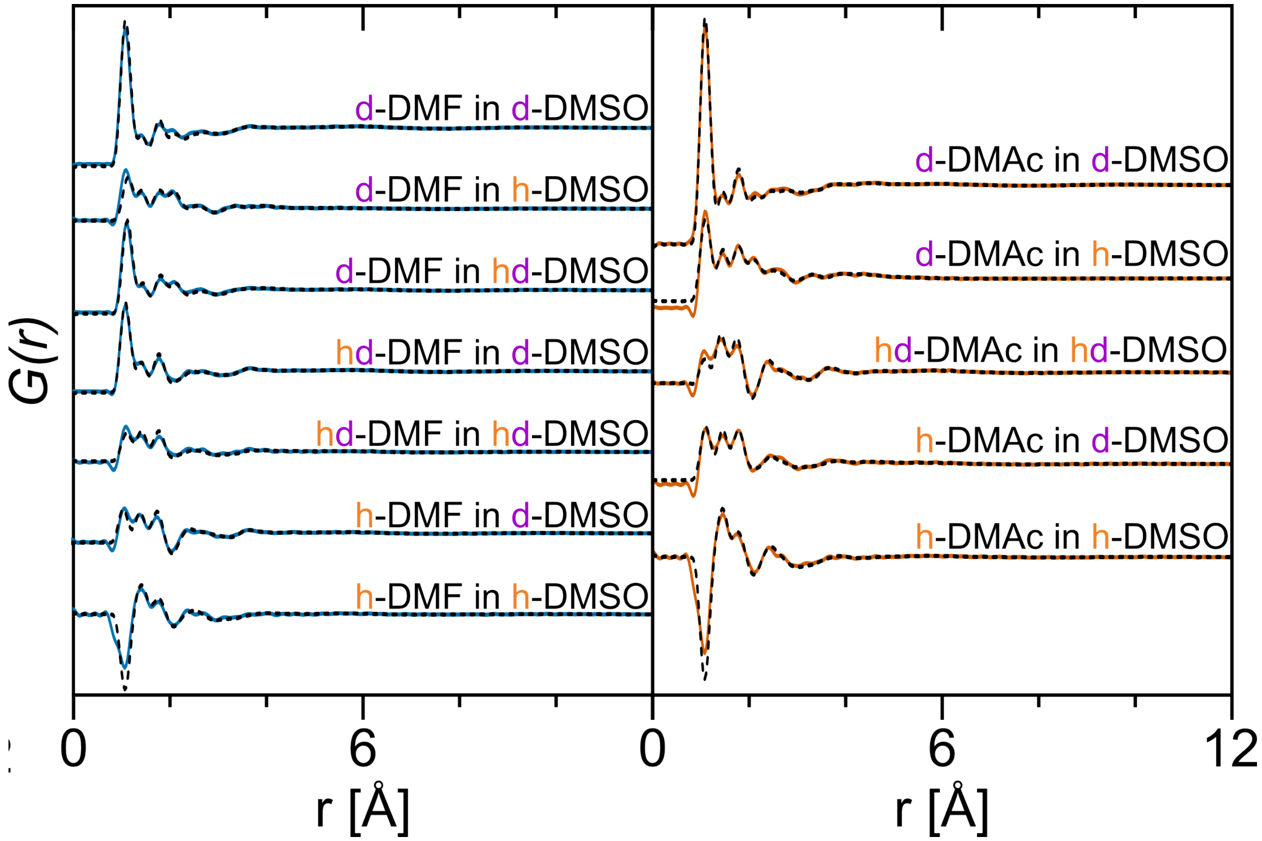

Figure S2: Experimental (solid–line) and modelled (dashed–line) total radial distribution functions, *G(r)* for DMF/DMSO equimolar mixture (left) and DMAc/DMSO equimolar mixture (right). Note the excellent agreement between the experimental and EPSR-generated total radial distributions in the region of the intramolecular bond distances.

S2: Molecular Bond Lengths and Angles

Table S1: Bond lengths and angles for DMF, DMAc and DMSO. The dihedral angles are set to maintain the tertiary amides planar, and to reflect the trigonal pyramidal molecular geometry of DMSO.[2–6]

		Bond Length /Å		Bond Angle /°		Dihedral Angle /°
DMF	H - C	1.11	H - C - O	123.60	H - C - N - C^Z	-0.160
	C - O	1.21	H - C - N	113.95	H - C - N - C^E	-178.27
	C - N	1.37	O - C - N	122.44	O - C - N -C^Z	179.80
	C^Z - N	1.46	C - N -C^Z	122.31	O - C - N - C^E	1.68
	C^E - N	1.46	C - N - C^E	121.73		
	H^Z–C^Z	1.11	C^Z - N - C^E	115.94		
	H^E–C^E	1.11				
DMAc	H^A–C^A	1.10	C^A - C - O	119.20	C^A - C - N - C^Z	0.0
	C^A - C	1.51	C^A - C - N	116.64	C^A - C - N - C^E	0.0
	C - O	1.23	O - C - N	124.16	O - C - N - C^Z	180.00
	C - N	1.40	C - N -C^Z	122.34	O - C - N - C^E	180.00
	C^Z - N	1.46	C - N - C^E	122.34		
	C^E - N	1.46	C^Z - N - C^E	115.33		
	H^Z–C^Z	1.09				
	H^E–C^E	1.09				
DMSO	O - S	1.50	O - S - C	107.50		
	S - C	1.81	C - S - C	95.76		
	H - C	1.09				

S3: Additional Partial Radial Distribution Functions *g(r)*

Partial Radial Distribution Functions, $g_{\alpha\beta}(r)$, are defined in Equations 2 and 3 of the main text. Here we present additional site–site combinations to supplement those in the main text Figures 6 and 7.

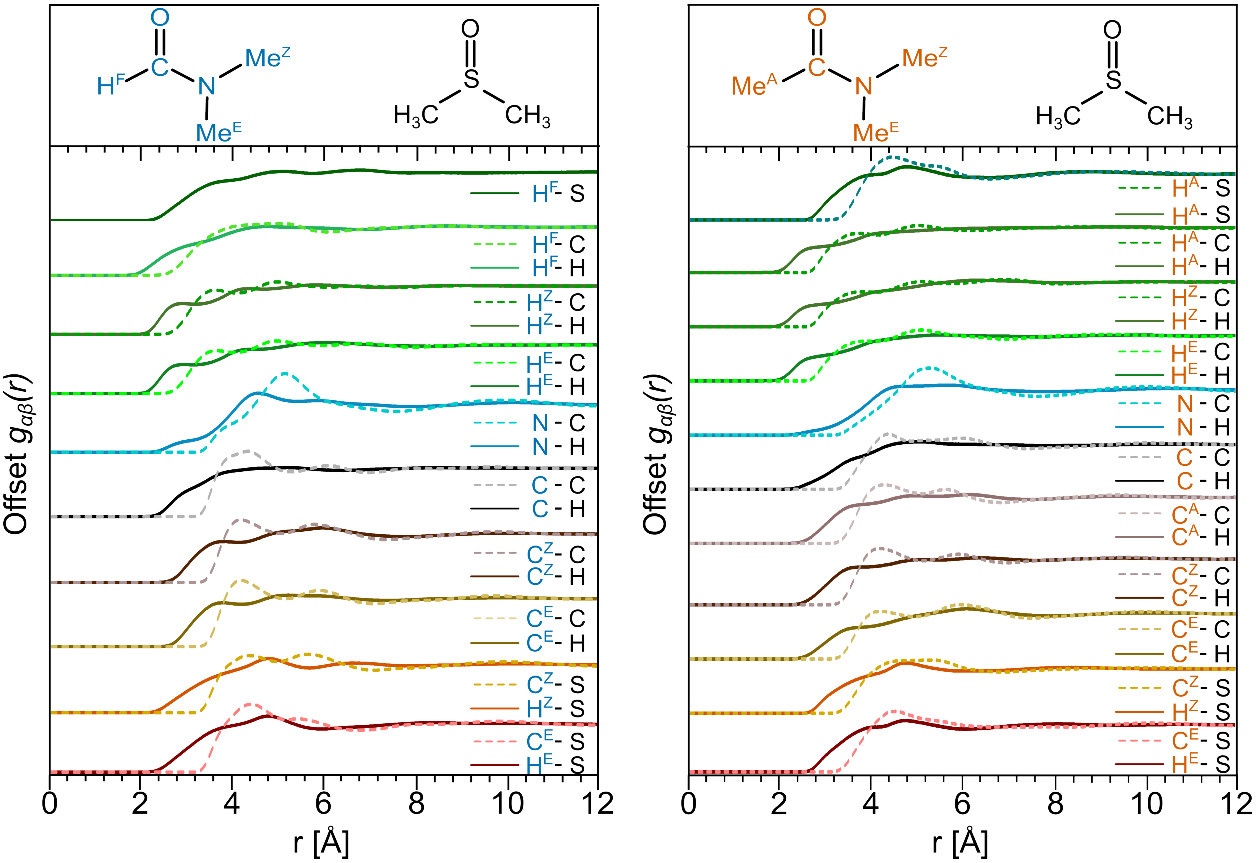

Figure S3: Inter–molecular Partial Radial Distribution Functions for the DMF/DMSO iteraction (left) and DMAc/DMSO (right). Note that the approach between DMF and DMAc with DMSO is very similar.[7]

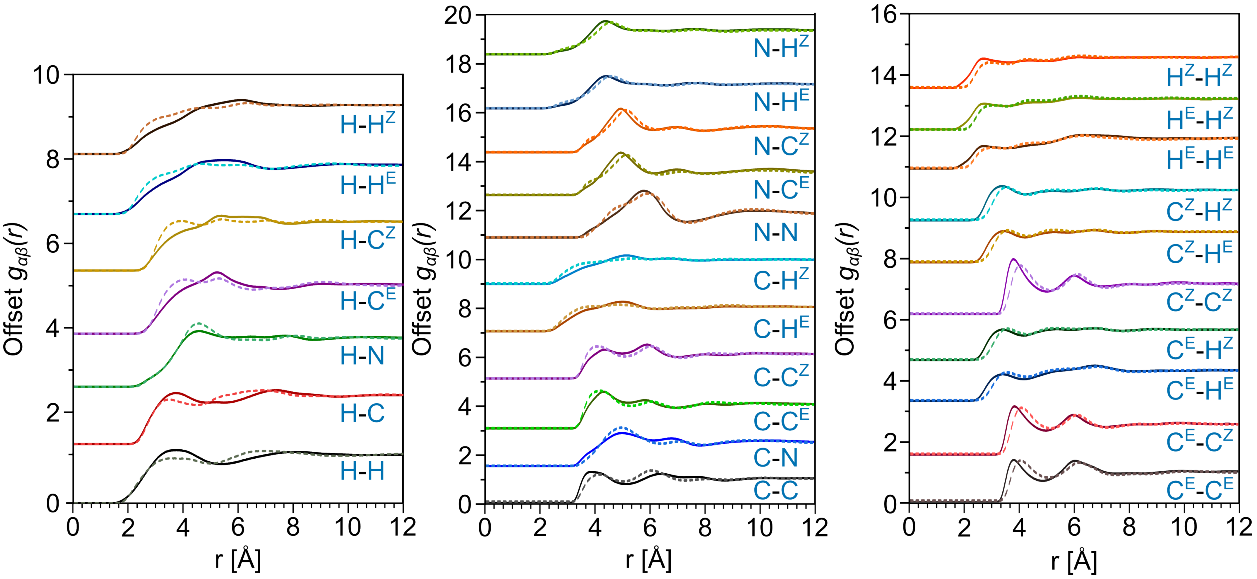

Figure S4: Inter–molecular Partial Radial Distribution Functions, $g_{\alpha\beta}(r)$, for DMF pure liquid (solid–line) compared to the structure of DMF in the presence of DMSO (dashed–line).[7] DMSO doesn't disrupt the local structure of the amide, proving its versatility as a co–solvent.

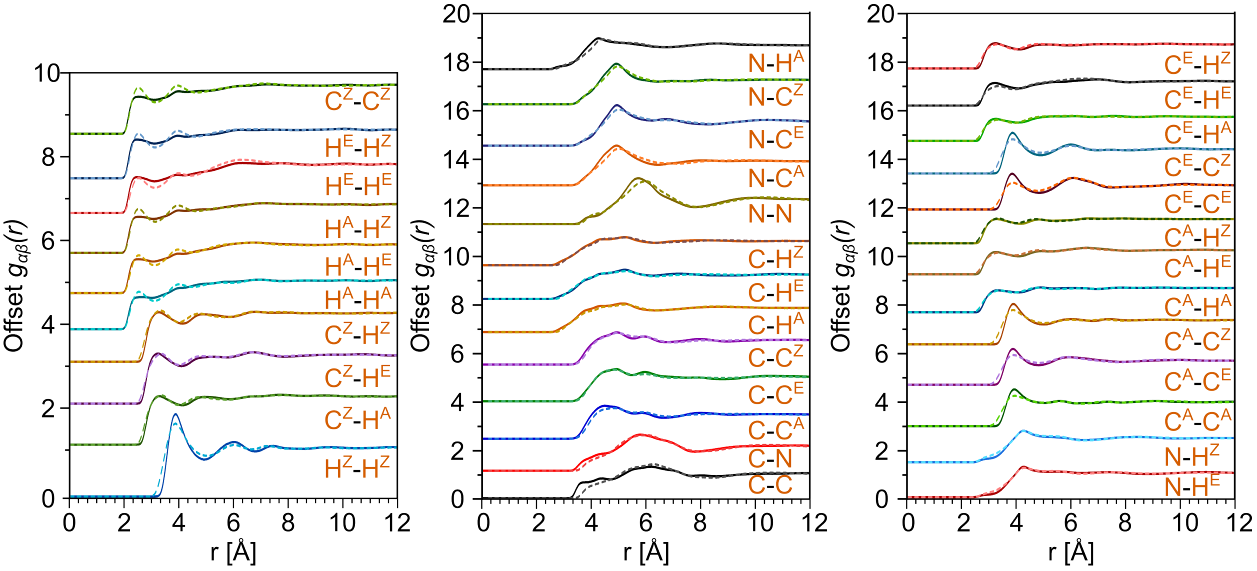

Figure S5: Inter–molecular Partial Radial Distribution Functions, $g_{\alpha\beta}(r)$, for DMAc pure liquid (solid–line) compared to the structure of DMAc in the presence of DMSO (dashed–line).[7]

S4: Intermolecular Partial Structure Factors $S_{\alpha\beta}(Q)$

Selected intermolecular partial structure factors were obtained according to Equation 2 of the main text.

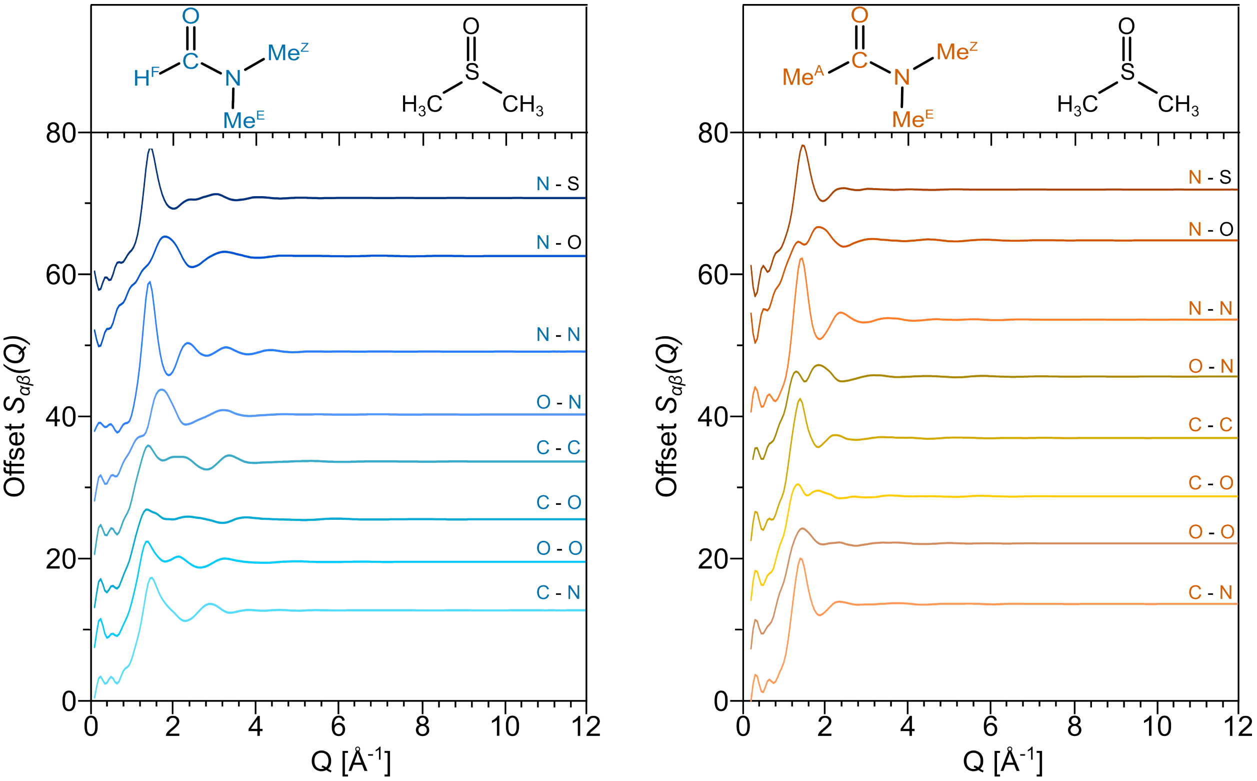

Figure S6: Selected inter–molecular partial structure factors $S_{\alpha\beta}(Q)$ for the DMF/DMSO (left) and DMAc/DMSO (right).

Section S5: Classical Monte Carlo Simulations

Monte Carlo simulations of the EPSR systems were conducted using the programme *Monte*.[8] A real-space cut-off of 12 Å was applied along with Ewald summation of long-range Coulombic interactions using a reciprocal-space cut off of 1.5 $Å^{-1}$ and convergence factor $\alpha = 0.25$.[9] Simulations were conducted at 290 K under the rigid molecule approximation. Simulation cell dimensions and compositions were identical to those used in the EPSR modelling: 700 molecules for pure liquids with cubic box size 44.80 Å for DMF and 47.58 Å for DMAc, and 1000 molecules for 50:50 mixed systems with cubic box 49.77 Å for DMF:DMSO and 51.63 Å for DMAc:DMSO. Inter-molecular interaction parameters were taken from the OPLS force-fields used as seed potentials for the EPSR simulations.[10–13] Simulation cells were equilibrated for approximately 5,000,000 iterations and data collection executed at 290 K over approximately 3,000,000 iterations. The CoM-CoM radial distribution functions and coordination numbers were calculated according to Equation 3 of the main text, and are compared with those obtained from EPSR fitting to the data in Figure S7 and S8. Table S2 shows the coordination numbers calculated to the same integration limits as those in Table 4 of the main text.

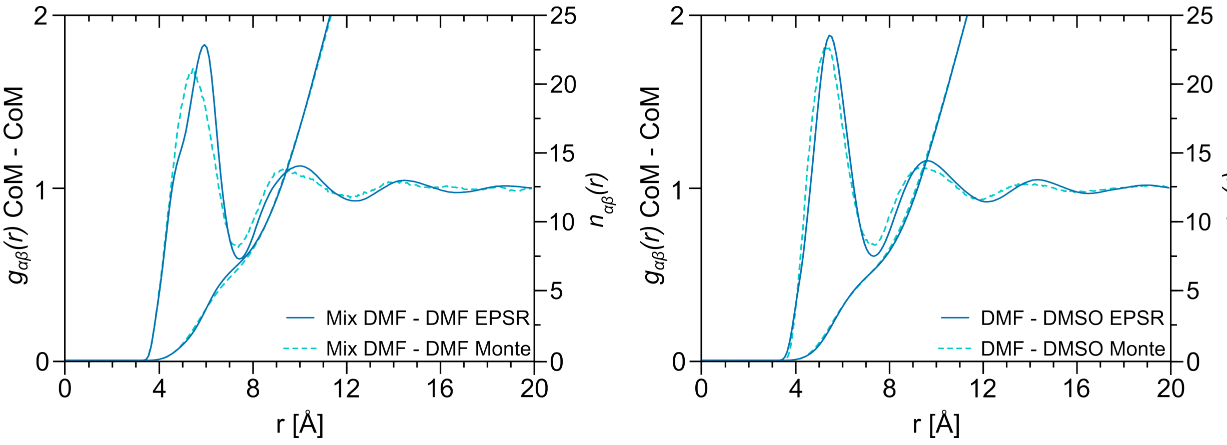

Figure S7: Comparison between the inter–molecular partial radial distribution functions for the DMF/DMF (left) and DMF/DMSO (right) in EPSR (solid-line) and Monte (dotted-line). The CoM-CoM interactions are very similar, EPSR is performing subtle changes to the structure in order to reproduce the experimental data.

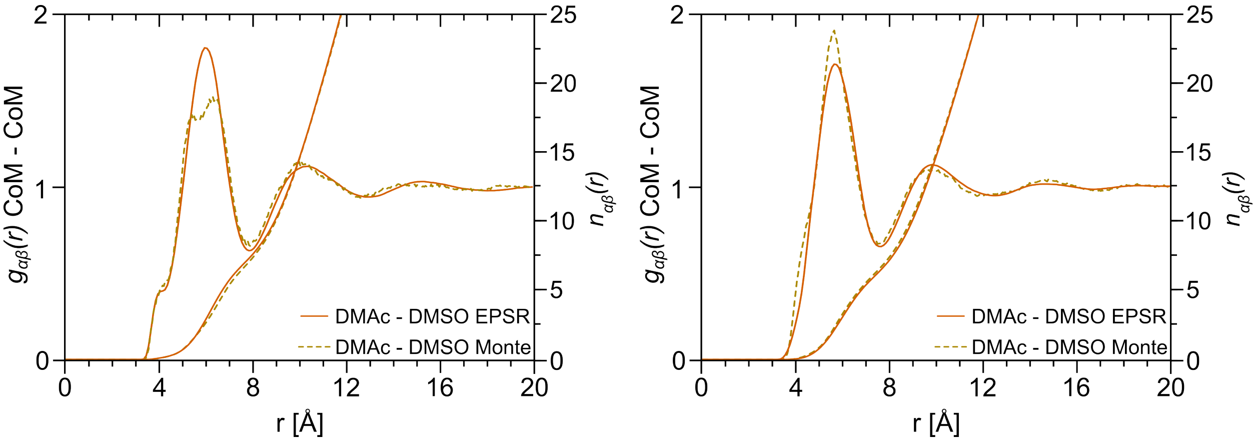

Figure S8: Comparison between the inter–molecular partial radial distribution functions for the DMAc/DMF (left) and DMAc/DMSO (right) in EPSR (solid-line) and Monte (dotted-line). The CoM-CoM interactions are very similar, EPSR is performing subtle changes to the structure in order to reproduce the experimental data.

Table S2: CoM-CoM first and second peak positions, integration limits and coordination numbers in the pure liquid amides and the 50 : 50 mixtures with DMSO calculated from classical Monte Carlo simulation using the programme *Monte*.[8]

	1st Peak /Å Pure/Mix	Integration Limit /Å Pure/Mix	Coordination Number (±0.1) Pure/Mix
**DMF – DMF**	5.50/5.50	7.46*/7.46*	13.1/6.8
**DMAc – DMAc**	6.15/6.30	7.87*/7.87*	13.1/7.1
**DMF – DMSO**	5.30	7.36	6.6
**DMAc – DMSO**	5.60	7.62	6.6
**DMSO – DMSO**	4.95	7.28	13.2

* The integration limit is set to the first minimum of the mix to allow more direct comparison between the corresponding coordination numbers.

References

(1) Squires, G. L. *Introduction to the Theory of Thermal Neutron Scattering*, 3rd ed.; Cambridge University Press, 2012.

(2) Adachi, S.; Kumagai, N.; Shibasaki, M. Conquering amide planarity: Structural distortion and its hidden reactivity. *Tetrahedron Lett.* **2018**, *59*, 1147–1158.

(3) Mujika, J. I.; Matxain, J. M.; Eriksson, L. A.; Lopez, X. Resonance structures of the amide bond: The advantages of planarity. *Chem. Eur. J.* **2006**, *12*, 7215–7224.

(4) Thomas, R.; Shoemaker, C. B.; Eriks, K. The molecular and crystal structure of dimethyl sulfoxide, $((H_3C)_2SO$. *Acta Crystallogr.* **1966**, *21*, 12–20.

(5) Halgren, T. A. Merck molecular force field. I. Basis, form, scope, parameterization, and performance of MMFF94. *J. Comput. Chem.* **1996**, *17*, 490–519.

(6) Hanwell, M. D.; Curtis, D. E.; Lonie, D. C.; Vandermeersch, T.; Zurek, E.; Hutchison, G. R. Avogadro: an advanced semantic chemical editor, visualization, and analysis platform. *J. Cheminformatics* **2012**, *4*, 1–17.

(7) Terban, M. W.; Billinge, S. J. Structural analysis of molecular materials using the pair distribution function. *Chem. Rev.* **2021**,

(8) Boek, E.; Coveney, P.; Skipper, N. Monte Carlo molecular modeling studies of hydrated Li-, Na-, and K-smectites: Understanding the role of potassium as a clay swelling inhibitor. *J. Am. Chem. Soc.* **1995**, *117*, 12608–12617.

(9) Wells, B. A.; Chaffee, A. L. Ewald summation for molecular simulations. *J. Chem. Theory. Comput.* **2015**, *11*, 3684–3695.

(10) Jorgensen, W. L.; Swenson, C. J. Optimized intermolecular potential functions for amides and peptides. Structure and properties of liquid amides. *J. Am. Chem. Soc.* **1985**, *107*, 569–578.

(11) Jorgensen, W. L.; Maxwell, D. S.; Tirado-Rives, J. Development and testing of the OPLS all-atom force field on conformational energetics and properties of organic liquids. *J. Am. Chem. Soc.* **1996**, *118*, 11225–11236.

(12) Zheng, Y.-J.; Ornstein, R. L. A molecular dynamics and quantum mechanics analysis of the effect of DMSO on enzyme structure and dynamics: subtilisin. *J. Am. Chem. Soc.* **1996**, *118*, 4175–4180.

(13) Yan, X. C.; Robertson, M. J.; Tirado-Rives, J.; Jorgensen, W. L. Improved description of sulfur charge anisotropy in OPLS force fields: model development and parameterization. *J. Phys. Chem. B* **2017**, *121*, 6626–6636.
